# Reverse total shoulder arthroplasty outcomes in elderly patients

**DOI:** 10.1016/j.jseint.2025.01.006

**Published:** 2025-02-06

**Authors:** Farah Selman, Brett Moreira, Nicholas P.J. Perry, Philipp Kriechling, Maximilian Gressl, Karl Wieser

**Affiliations:** a Department of Orthopedics, Balgrist University Hospital, Zurich, Switzerland; b Orthopaedic Department, University Hospital Geelong, Geelong, Victoria, Australia; c Department of Orthopedic Surgery, Naval Medical Center San Diego, San Diego, CA, USA

**Keywords:** rTSA, Reverse total shoulder arthroplasty, Clinical and radiographic outcome, Elderly, Age, Complications

## Abstract

**Background:**

Previous studies have found varied outcomes for elderly patients following reverse total shoulder arthroplasty (rTSA). Results for very old patients are rare. The purpose of this study was to analyze functional and radiographic outcomes after rTSA in patients aged 85 years and older and to compare them to a younger population.

**Methods:**

Of 1460 patients treated with rTSA from 2005 to 2020, 5% (n = 74) were ≥85 years. 32 patients were excluded due to travel difficulties, death, refuse to participate and lost to follow-up. 42 patients with a minimum follow-up of 2 years were included and matched for sex, body mass index, American Society of Anesthesiologists Score, surgical indication, smoking, alcohol consume, and follow-up time to a younger population with a mean age of 69.5 ± 9 years. Statistical analysis was performed based on preoperative and postoperative range of motion (ROM), pain scores and patient satisfaction. Postoperative X-rays were evaluated for notching, radiolucency, bone formation and resorption, implant migration and periprosthetic fractures.

**Results:**

42 cases with a mean age of 87 ± 2 years were included, 71% female. Indications for rTSA were rotator cuff (RC) tear with arthritis (36%), RC arthropathy (17%), RC tear without arthritis (17%), primary arthritis (17%), proximal humeral fracture (10%), failed osteosynthesis (7%), and instability (2%). Mean follow-up was 47 ± 22 months. Except for age, there were no significant differences in the matching cohort. In the elderly cohort, relative Constant-Murley Score (CMS) improved postoperative significantly from 41 ± 20 to 83 ± 11 and subjective shoulder value from 38 ± 20 to 80 ± 19. All aspects of ROM, except for external rotation, improved significantly. Abduction force and pain improved as well. Radiolucency around the glenoid (21%), bone formation (4.9%) and implant migration (4.9%) were significantly associated with poorer outcome in the absolute CMS (*P* = .013, *P* = .002 and *P* = .008). One patient (1/42, 2.4%) suffered multiple shoulder dislocations and needed a revision. No other reoperations were performed. Comparison between patients over 85 years and the younger matched control group showed no significant differences in relative CMS, subjective shoulder value, and ROM at final follow-up.

**Conclusion:**

Patients aged 85 years and older can expect significant improvement in shoulder function and significant reduction of pain after rTSA. Overall clinical outcome is comparable to a younger population. Complication and revision rates are low. Age should not be a limiting factor when considering rTSA.

The worldwide aging population^4^^,^^16^ following advances in all fields of medicine has led to an increased demand for orthopedic treatments that allow elderly people to not only perform activities of daily living but also to participate in sports. As such, rotator cuff (RC) tears and other degenerative and traumatic shoulder pathologies have become a growing issue in patients over 80 years^49^ and research has focused on delivering valid therapeutic options to restore mobility in these individuals. The reverse total shoulder arthroplasty (rTSA), developed in 1987 by Grammont et al,^26^ now plays an essential role in the treatment of various shoulder lesions.^6^^,^^27^

The indications for rTSA range from irreparable RC tears to osteoarthritis and rheumatoid arthritis as well as comminuted fractures of the humeral head.^58^ There are various studies highlighting the benefits of rTSA when revision of primary anatomic shoulder arthroplasty is performed.^40^^,^^45^^,^^50^ In addition, there is an indication for rTSA in patients with a history of severe instability due to glenoid bone loss.^66^

Due its use in the conditions described above, rTSA has gained popularity in recent years,^6^^,^^17^^,^^65^ although there are complications that can arise from its application.^37^ Their incidence depends on the respective indication for surgery and varies between 15% and 50%.^37^^,^^67^ Periprosthetic fracture, glenoid or humeral loosening, notching, instability and infection are some of the intraoperative and postoperative events that can have significant impact on patient satisfaction.^34^^,^^37^^,^^53^

Despite former belief suggesting that rTSA should be reserved to elderly patients with lower functional demand,^22^^,^^29^ rTSA is nowadays also used in the younger patient cohort (<65 years).^9^^,^^11^^,^^12^^,^^21^^,^^51^^,^^54^ Comparisons between the outcomes of the younger and older population vary in literature, some favorizing older population,^25^^,^^48^^,^^60^ some the younger.^19^^,^^35^ In general, there is no consensus regarding the outcome of rTSA in very old patients (≥80 years).^1^^,^^2^^,^^7^^,^^13^^,^^15^^,^^18^^,^^36^^,^^42^^,^^61^^,^^63^

The aim of this study was to evaluate clinical, functional as well as radiologic features of patients over 85 years who have undergone rTSA and to compare these results with a younger population. Our hypothesis is that clinical and radiological outcomes after rTSA in patients aged 85 years or older are comparable to those in a younger group.

## Methods

Before initiation of the study, approval was obtained from the responsible regional ethical review board (BASEC ID 2018-01494).

### Patients

Between September 2005 and November 2020, 1460 patients were treated with rTSA at our hospital. Their preoperative and postoperative records were saved in an institutional registry.

**Figure 1:**
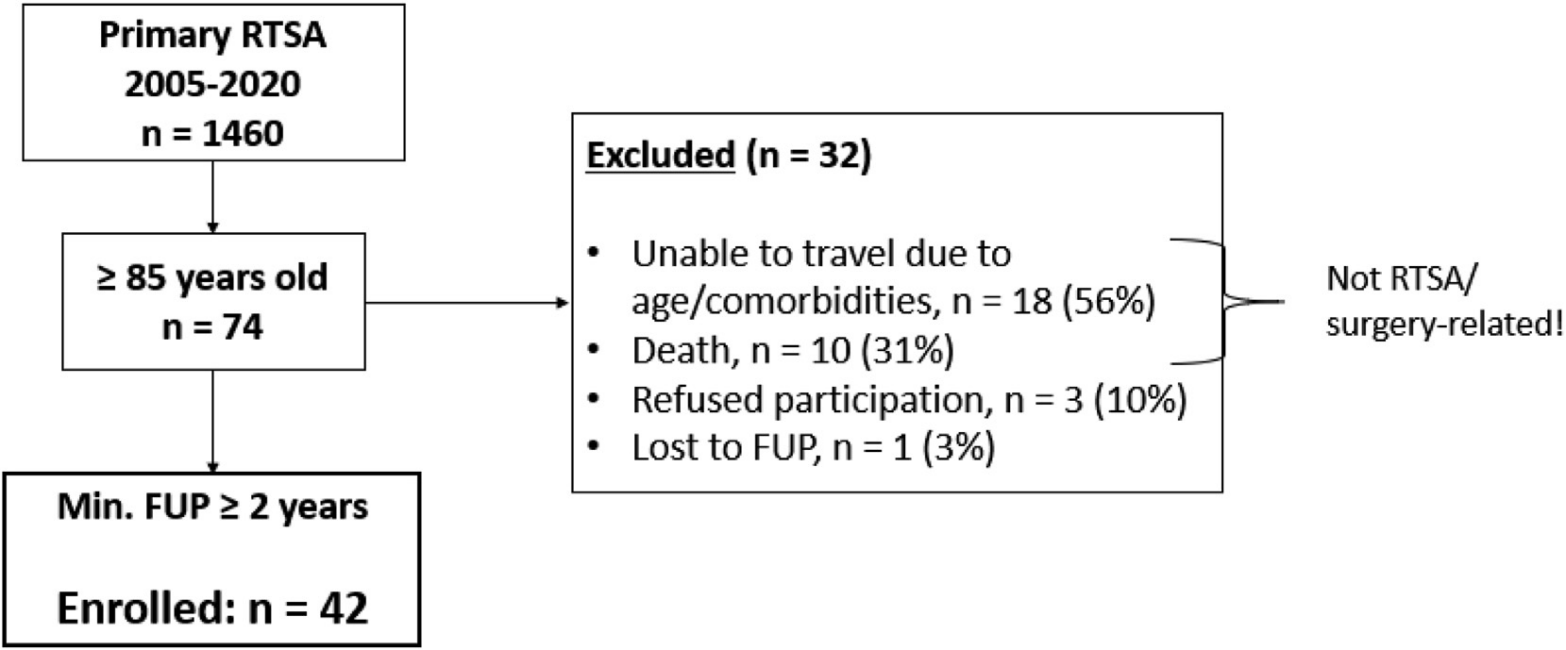
Flowchart of patient inclusion/exclusion. *RTSA*, reverse total shoulder arthroplasty; *FUP*, follow-up.

By retrospective review, we identified 74 patients (5%) aged 85 years and above. Of those, 32 patients were excluded – 18 due to travel difficulties (age and comorbidity related); 10 due to (treatment unrelated) death (Table I); 3 patients refused to participate in the study and 1 patient was lost to follow-up. None of the exclusions were rTSA/surgery related. The remaining 42 patients had completed a minimum follow-up period of 2 years and were therefore defined as the final sample-size (Fig. 1).

**Table I:** Baseline demographics of cases and controls.

	Cases (N = 42)	Controls (N = 42)	*P* value
Age at surgery [y]	87 (85.7, 87.8)	72.8 (71.0, 73.6)	<.001∗
Gender [female]	71% (30)	62% (26)	.4
Body mass index [kg/m^2^]	25.1 (23.4, 27.0)	25.0 (23.4, 28.0)	.6
ASA-classification, (%)			
I	0 (0)	1 (2.4)	.6
II	19 (45)	22 (52)	
III	22 (52)	19 (45)	
IV	1 (2.4)	0 (0)	
Smoking, (%)			
Never	36 (86)	33 (79)	.7
Stopped	5 (12)	7 (17)	
Unknown	1 (2.4)	2 (4.8)	
Alcohol, (%)			
Unknown	3 (7)	3 (7)	>.9
Never	20 (48)	19 (45)	
Rarely	12 (29)	13 (31)	
Regularly	5 (12)	6 (14)	
Abuse	2 (5)	1 (2.4)	
Indication, (%)			
RCT with OA	14 (33)	15 (36)	.8
CTA	7 (17)	3 (7.1)	
RCT without OA	6 (14)	7 (17)	
Primary OA	6 (14)	9 (21)	
Fracture	4 (9.5)	4 (9.5)	
Conversion plate	3 (7)	2 (5)	
Instability	1 (2)	2 (5)	
Follow-up [mo]	54 (26, 60)	45 (24, 60)	.3
Side of the shoulder [right], (%)	26 (62)		
Number of previous surgeries, (%)			
0	31 (74)		
1	10 (24)		
2	1 (2)		
≥3	0 (0)		

*ASA*, American Society of Anesthesiologists; *CTA*, cuff tear arthropathy; *RCT*, rotator cuff tear; *OA*, osteoarthritis.

All values are given in absolute number (percentage of total). Median (interquartile range).

∗ Marks significance of the *P* value.

**Figure 2:**
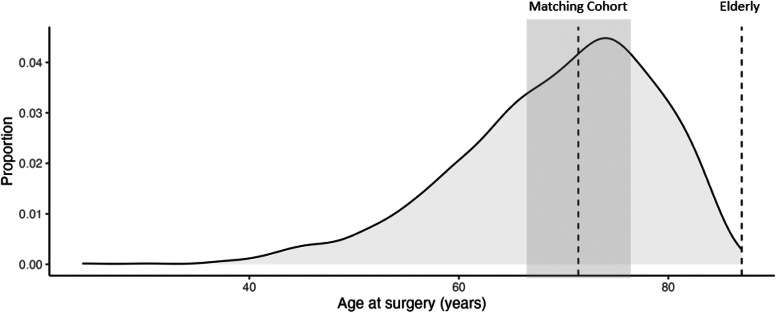
Age distribution of patients with rTSA. *Dashed lines*: Medians of the younger and elderly population. *Grey area*: matched cohort consisting of the median age of our rTSA Database ± 9 years. *rTSA*, reverse total shoulder arthroplasty.

In a second step, we looked at the age distribution in our rTSA database (Fig. 2). We took the median age of 69.5 years as a starting point with a range of ± 10 years as possible matching cohort (Fig. 2). Out of this population, a 1:1 matching according to Sex, body mass index, American Society of Anesthesiologists (ASA) score, surgery indication, smoking, alcohol consume, and follow-up time was conducted. 42 patients with a mean age of 69.5 ± 9 years represented our control group.

### Surgical technique

The surgeries were performed by specialized shoulder and elbow surgeons through a deltopectoral approach as previously described. All surgeons used the Zimmer Reverse Anatomical Shoulder System (Zimmer Biomet, Warsaw, IN, USA). According to the indication, different rTSA stem designs were fitted, mostly with an onlay configuration of the polyethylene cup and a neck-shaft angle of 135 degrees. Depending on bone quality and quality of the press fit, additional stem cementation was performed.

### Data collection and outcome measures

The clinical preoperative as well as follow-up examinations of each patient were performed following a standardized assessment protocol. The Constant-Murley Score (CMS)^14^ was calculated based on pain, range of motion (ROM; flexion, abduction, external, and internal rotation) as well as mean force in abduction. Subjective shoulder value (SSV)^24^ was also determined preoperative and postoperatively. Information was documented in our data processing system Research Electronic Data Capture (Research Electronic Data Capture; Vanderbilt University, Nashville, TN, USA).^31^^,^^32^ In addition to the clinical findings, assessment of radiographic imaging was performed in each patient. The 6 weeks, 4.5 months, 1-year, and latest (≥2 years) postoperative follow-up radiographic images were evaluated by a musculoskeletal radiologist and the two authors for signs of notching, glenoid and humeral lucency, heterotopic ossification and implant migration. Subclassification was performed for notching according to the Nerot-Sirveaux-Classification^57^ from grade 1 to grade 4, glenoid and humeral lucency according to Sperling et al^59^ and for heterotopic ossification in compliance with the modified Brooker classification^64^ into grade 1a, 1b, 1c, and 2.

### Statistical analysis

Statistical analysis was performed using the software RStudio (version 2022.12.0, Build 353; Posit, Boston, MA, USA). The acquired data was assessed for normality using the Shapiro-Wilk test, parametric variables were tested with Student-t test and nonparametric variables were tested using the Mann-Whitney U test or Wilcoxon test to detect significant differences between clinical findings at a preoperative and postoperative examination as well as immediate postoperative and follow-up X-ray images. Multivariate logistic regression analysis for outcome predictors was calculated using the final fit module. For *P* values less than .05, statistical significance was assumed.

## Results

### Baseline demographics

Among the 42 cases mean age at surgery was 87 ± 2 years, dominantly females (71%) and mean follow-up period of 47 ± 22 months. The main indications for rTSA were irreparable RC tear with osteoarthritis (33%) and cuff tear arthropathy (17%). Nineteen patients (45%) were classified according to the "American Society of Anesthesiologists Physical Status Classification System"^33^ as ASA 2, 22 (52%) as ASA 3, and one case (2%) as ASA 4.

There were no significant differences between the cases and controls according to Sex, body mass index, ASA score, surgery indication, smoking, alcohol consume, and follow-up time.

Detailed information on baseline demographic data can be found in Table I.

### Lost to follow-up and mortality

All patients who were primarily not available for the standard 2-year follow-up were contacted to evaluate the cause of lost to follow-up. Of the 74 patients older than 85 years, a total of 10 patients (31%) died during the 2 years of follow-up period. All deaths were unrelated to the rTSA implantation and beyond the immediate postoperative period, defined as >90 days postoperative (Table II).

**Table II:** Time from shoulder arthroplasty to patient’s death.

Patient number	Age at surgery [y]	Shoulder arthroplasty to death [mo]
1	85.3	68.5
2	86.8	15.1
3	85.9	3.1
4	94.0	30.6
5	88.4	15.1
6	87.8	15.1
7	87.9	22.6
8	87.5	31.9
9	85.6	29.0
10	85.8	8.1
Mean ± standard deviation	87.5 ± 2.5	23.9 ± 18.4

### Complications and revisions

A total of 6 (14.2%) intraoperative complications in the sense of nondisplaced humeral fissures occurred in the elderly group. 2 of them (33.3%) were protected with wire cerclage, 4 (66.6%) were with FiberWire cerclage (Arthrex, Naples, FL, USA). During hospitalization no major medical complications occurred.

One (2.4%) patient suffered an anterior dislocation while lifting a heavy object 3 months postoperatively, which required closed reduction. After further instability with multiple dislocations, a revision surgery was performed 5 months postoperatively. No other revisions were needed.

### Clinical outcome

Patient satisfaction, ROM and CMS could be significantly improved by the treatment with rTSA in the elderly group. The SSV increased from 38% preoperatively to 80% at 2-year follow-up. Flexion and abduction grades have also almost doubled (67°-116°, 64°-118°). Only the external rotation could not be improved significantly with an rTSA (21°-24°, *P* = .29). Relative CMS improved from 41 to 83 points postoperative.

At last follow-up there were no significant differences in the clinical outcome between cases and the younger controls.

A detailed overview of all outcome scores can be found in Tables III and IV.

**Table III:** Outcome scores and ROM in the elderly group preoperative and at 2-year follow-up.

	Preoperative	2-year FUP	*P* value
Constant score, absolute	29 ± 15	64 ± 10	<.01∗
Constant score, relative	41 ± 20	83 ± 11	<.01∗
Subjective shoulder value [%]	38 ± 20	80 ± 19	<.01∗
CS pain	7 ± 4	14 ± 2	<.01∗
Flexion [°]	67 ± 35	116 ± 23	<.01∗
Abduction [°]	64 ± 33	118 ± 23	<.01∗
External rotation [°]	21 ± 21	24 ± 20	.29
Internal rotation [points]	3 ± 3	5 ± 3	<.01∗
Mean force [kg]	1 ± 2	2 ± 2	.01∗

*FUP*, Follow-up; *CS*, constant score.

All values are given in mean ± standard deviation.

∗ Marks significance of the *P* value.

**Table IV:** Outcome scores and range of motion in cases and controls at last follow-up.

Last FUP	Cases (N = 42)	Controls (N = 42)	*P* value
Constant score, absolute	67 (58, 71)	69 (63, 76)	.14
Constant score, relative	85 (75, 90)	81 (75, 90)	.6
Subjective shoulder value [%]	90 (70, 92)	90 (75, 95)	.7
CS pain	15 (15, 15)	15 (13.5, 15)	.5
Flexion [°]	120 (110, 130)	123 (115, 134)	.15
Abduction [°]	120 (103, 130)	130 (103, 152)	.061
External rotation [°]	25 (10, 30)	30 (20, 50)	.056
Internal rotation [points]	6 (4, 8)	6 (4, 8)	.5
Mean abduction force [kg]	1.97 (0, 2.73)	2.60 (0.13, 3.47)	.2

*FUP*, Follow-up; *CS*, constant score.

All values are given in median (interquartile range).

### Radiographic assessment

At 2-year follow-up 59.5% of the elderly patients did not have signs of scapular notching. 7 patients (16.7%) had grade 1 or 2 notching, 4 patients (9.5%) grade 3, and 6 patients (14.3%) grade 4 notching. Bone formation, mostly type 1b (57.1%), was observed in 64.3% of patients. Radiological overall glenoid lucency was seen in 21.4% and humeral lucency in different zones in 36.6% of patients. Only 2 patients (4.9%) had signs of implants migration. A detailed overview with a comparison of radiographic parameters directs postoperatively and 2-year postoperatively can be found in Table V.

**Table V:** Radiographic outcomes 6 weeks and 2 years postoperatively in the elderly group.

	Total examined, (%)	Grade	Total of grade, (%)	6 weeks postoperative, (%)	2-year FUP, (%)	*P* value
Notching	84 (100)	None	65 (77.4)	40 (95.2)	25 (59.5)	.002∗
		1	7 (8.3)	1 (2.4)	6 (14.3)	
		2	2 (2.4)	1 (2.4)	1 (2.4)	
		3	4 (4.8)	0	4 (9.5)	
		4	6 (7.1)	0	6 (14.3)	
Glenoid lucency	84 (100)	None	74 (88.1)	41 (97.6)	33 (78.6)	.018∗
		1	10 (11.9)	1 (2.4)	9 (21.4)	
Humeral lucency	83 (98.8)	none	68 (81.9)	42 (100)	26 (63.4)	<.001∗
		1	15 (18.1)	0	15 (36.6)	
Bone formation	83 (98.8)	None	55 (66.3)	40 (97.6)	15 (35.7)	<.001∗
		1a	3 (3.6)	1 (2.4)	2 (4.8)	
		1b	24 (28.9)	0	24 (57.1)	
		1c	1 (1.2)	0	1 (2.4)	
Implant migration	82 (97.6)	None	80 (97.6)	41 (100)	39 (95.1)	.474
		1	2 (2.4)	0	2 (4.9)	

*FUP*, follow-up.

All values are given in absolute number (percentage of total).

∗ Marks significance of the *P* value.

Loosening of the glenoid component, bone formation grade 1a and implant migration were significantly associated with worse patient outcomes as seen in the mean CMS (*P* = .013, *P* = .002, *P* = .008). Notching of any grade, lucency of the humeral component and bone formation grade 1b showed no statistically significant effect on 2-year-outcomes.

Details can be found in Table VI.

**Table VI:** Radiographic factors with influence on clinical outcome in the elderly group at 2-year follow-up.

2 y FUP	Total examined, (%)	Grade	Total of grade, (%)	Mean CS	OR	*P* value
Notching	42 (100)	None	25 (61.0)	67.5 (±9.2)		
		1	6 (14.6)	61.5 (±7.1)	5.98 (±9.16)	.194
		2	1 (2.4)	No analysis	No analysis	-
		3	4 (9.8)	60.0 (±9.5)	7.48 (±10.85)	.171
		4	6 (14.6)	59.5 (±14.9)	7.98 (±9.16)	.086
Glenoid lucency	42 (100)	None	33 (78.6)	66.4 (±9.4)		
		1	9 (21.4)	57.0 (±10.7)	9.39 (±7.34)	.013∗
Humeral lucency	41 (97.6)	None	26 (63.4)	62.8 (±11.3)		
		1	15 (36.6)	66.9 (±8.5)	4.02 (±6.79)	.239
Bone formation	42 (100)	None	15 (36.6)	65.0 (±11.1)		
		1a	2 (4.9)	42.0 (±12.7)	23.00 (±14.15)	.002∗
		1b	24 (58.5)	65.7 (±7.8)	0.67 (±6.18)	.829
Implant migration	41 (97.6)	None	39 (95.1)	64.9 (±9.3)		
		1	2 (4.9)	46.0 (±11.3)	18.92 (±13.73)	.008∗

*FUP*, follow-up; *CS*, constant score; *OR*, odds ratio.

All values are given in absolute number (percentage of total).

∗ Marks significance of the *P* value.

Comparison between cases and controls in the last follow-up shows significant differences in notching and new bone formation (Table VII). There were no significant differences in glenoid and humeral lucency and implant migration between the older and younger population (Table VII).

**Table VII:** Radiographic outcome of cases and controls at last follow-up.

Last FUP	Cases (N = 42), (%)	Controls (N = 42), (%)	*P* value
Notching			
None	25 (60)	22 (52)	<.001∗
1	6 (14)	14 (33)	
2	1 (2.4)	6 (14)	
3	4 (9.5)	0 (0)	
4	6 (14)	0 (0)	
Glenoid lucency	9 (21)	7 (17)	.6
Humeral lucency	15 (37)	13 (31)	.6
Bone formation			
None	15 (36)	35 (83)	<.001∗
1a	2 (4.8)	4 (9.5)	
1b	24 (57)	0 (0)	
1c	1 (2.4)	0 (0)	
2	0	3 (7.1)	
Implant migration	2 (4.9)	4 (9.5)	.7

*FUP*, follow-up.

All values are given in absolute number (percentage of total).

∗ Marks significance of the *P* value.

## Discussion

Our study showed low complication and revision rates, and a significant improvement in shoulder function and pain in patients older than 85 years after rTSA for different indications. These results were comparable to a matched controlled cohort of a younger patient population.

### Patient satisfaction

Overall, there seems to be a tendency towards higher patient satisfaction after rTSA in the elderly population.^3^^,^^43^^,^^44^^,^^48^^,^^60^ Neel et al^48^ show that patients aged <60 years had lower satisfaction scores (86% vs. 92%, *P* = .006) compared to elderly of 60-79 years. Stewart el al^60^ reported highest patient satisfaction in the age group of 70-79 years at 26 months follow-up. These patients reached almost 80 points in the SANE score, whereas patients <60 years only reached a mean score of 70 (*P* = .019). This observation could be explained by a lower demand in older patients. Although, Simovitch et al^55^ report a median SSV improvement from 27% preoperatively to 90% postoperatively (*P* ≤ .001) after rTSA. They analyzed a senior (mean age = 73 years) athletic population, which contradict the high satisfaction rate due to low demand.

Overall, these outcomes are similar to our absolute outcomes with mean SSV of 80% at 2-year follow-up. Comparing our cases with the younger controls at last follow-up, we could not show a significant difference in SSV. Both showed high satisfaction after rTSA (median 90%). It is conceivable that the age difference in our cohort was too small to show a significant difference.

In comparison to a higher age difference through a study conducted by Ernstbrunner el al^21^ in our institution with a mean age of 57 years, a difference could be seen. Their cohort showed a mean postoperative SSV of 71% ± 27%. This comparison may support Stewarts^60^ and Neels^48^ conclusion for a higher satisfaction in the older population compared to a population younger than 60 years.

### Functional outcome

Torrens et al^62^ suggested a minimal clinically important difference of 8 points in the absolute Constant Score. Our cohort had 35 points difference preoperative and postoperatively. These results are aligned with a systematic review, which investigated over 5800 shoulders of all ages (34-93 years) before and after primary rTSA and found a mean improvement of the absolute Constant Score by 37 points.^23^ Multiple other studies have also shown significant improvement in shoulder function after rTSA in the elderly population.^7^^,^^13^^,^^15^^,^^28^^,^^36^ Especially in the treatment for high-grade proximal humeral fractures, elderly patients with rTSA seem to have better functional outcomes compared to those treated with plate fixation.^8^^,^^38^^,^^41^^,^^56^

But also younger patients seemed to benefit significantly from the prosthetic treatment in terms of function.^9^^,^^21^^,^^30^^,^^47^^,^^52^^,^^55^^,^^60^ Samuelsen,^52^ Hanisch^30^ and Monir et al^47^ showed significant improvement in pain, ROM and strength for patients younger than 65 years after rTSA. In comparison to an older population, there seems to be a tendency for poorer outcome at early follow-up in the younger groups.^5^^,^^30^ However, these differences balanced out in long-term. Ek^20^ and Ernstbrunner et al^21^ documented good results up to and even beyond 10 years postoperatively. Frequently expressed concerns that a higher activity level in younger patients leads to excessive wear and therefore poor outcome^22^^,^^29^ can thus be presumably contradicted.

### Complications, revisions, and mortality

Our cohort of ≥patients aged 85 years showed a very low complication and revision rate. One patient (2.4%) suffered an anterior dislocation while lifting a heavy object 3 months postoperatively. Due to further instability, a revision was performed 5 months postoperatively. Despite some nondisplaced intraoperative fissures of the humeral stem which were protected by cerclage or suture cerclages and did not negatively influence patient outcome, there were no other medical or surgical complications and no other revisions. We lost 10 patients (31%) due to death, but none of those deaths were thought to be directly related to the patient’s shoulder surgery.

Clark et al^13^ reported a medical complication rate of 3% and a surgical complication rate of 12% in a cohort of 179 patients ≥80 years. Chamberlain et al^9^ reported the same overall complication rate of 12% in a younger cohort with a mean age of 54.8 years. Hanisch et al^30^ compared 2 cohorts over and under the age of 65 years. After 1 year, they found no differences in complications or revisions between both groups.

Testa et al^61^ conducted a national database analysis of patients 80 years and older and showed low complication and revision rates (1 year 3.9%, 2 year 5.1%). Nevertheless, patients 80 years and older had a significantly higher 90-day mortality rate compared with younger patients (2.7% and 1.5%, *P* = .002), but lower rates of dislocation, periprosthetic fracture, and implant-related complication at 1 year postoperatively. Their study is in line with our study, both of which suggest that primary rTSA in elderly patients is a safe procedure with few complications compared to rTSA in a younger cohort.

### Radiological findings

Scapular notching is a common finding in rTSA and well documented, but its clinical relevance is controversial. Some authors reported no influence on the outcome,^39^ others correlated notching with worse ROM and higher complication rates.^46^

Reviewing our postoperative radiographs, we identified notching of any grade in 39% (n = 19) of the shoulders at an average follow-up of 47 months. In a cohort of 171 patients aged 80 years and older, Kriechling et al^36^ reported notching in 40% of patients at a mean follow-up of 41 ± 25 months. These rates are relatively low compared to the incidence identified by Ernstbrunner et al,^21^ which was 95% two to five years postoperatively or Werner et al^66^ with 96% at 38 months follow-up. Chaudhury et al^10^ documented 21% of scapular notching at 2-year follow-up in their cohort. Monir et al^46^ analyzed patients under 65 years with rTSA. They had 9.6% (n = 5) scapular notching at 5-year follow-up, one of whom required revision arthroplasty. These results highlight the variation in reported rates of scapular notching at midterm follow-up. By subjectively analyzing notching rates and rCS's included in our cohort, we could not show a clear association between those two parameters. It’s important to mention, that notching is often described and analyzed as a binary variable, which ignores the grading and its possible effect on the outcome. Further subgroup analyses in this regard are desirable. In addition, several other factors such as implant size and configuration possible have a greater influence of the presences of scapular notching compared to patient age.

Other factors like humeral or glenoid lucency or heterotopic ossification have been rarely analyzed at mid- and long-time follow-up. We documented 21.4% (n = 9) glenoid and 36.6% (n = 15) humeral lucency in our cohort. And found a significant correlation of radiographic glenoid lucency (*P* = .013), heterotopic ossification grade 1a (*P* = .002) and implant migration (*P* = .008) with worse outcomes in terms of pain and function in our patient group.

### Limitations

All patient data of our study derives from a single institution with patients treated by a limited number of surgeons. However, the surgeons involved in performing the rTSA were all fellowship trained senior shoulder surgeons, which removes the confounding factor of low surgeon experience. A further limitation was the follow-up-period, with an average of 47 months. Longer term follow-up would have been ideal to assess clinical relevance of some of our radiographic findings. However, previous studies have commented on a lack of radiographic changes post-rTSA more than 10-year follow-up.^21^

This paper utilized consistent rTSA implant in all cases, with a high proportion of cementless humeral stems used in this age group (62%). Bias was minimized during clinical assessment and X-ray-evaluation as both tasks were performed independently by the researching team away from the operating surgeon.

A further limitation of our study is the relatively small cohort of elderly patients (85+ years) compared to other shoulder arthroplasty cohorts. However, this reflects an inherent limitation, as patients aged 85+ years represent a naturally smaller subset of the overall rTSA population.

## Conclusion

Our study demonstrates a low complication and revision rate and a significant improvement in shoulder function for patients over 85 years undergoing rTSA. Therefore, age over 85 years should not be considered as a contraindication for rTSA, when the demand for improved shoulder function is required and improvement in quality of life, regardless of the underlying pathology or diagnosis.

## Disclaimers:

Funding: No funding was disclosed by the authors.

Conflicts of interest: The authors, their immediate families, and any research foundation with which they are affiliated have not received any financial payments or other benefits from any commercial entity related to the subject of this article.
